# Understanding the experience and manifestation of depression in adolescents living with HIV in Harare, Zimbabwe

**DOI:** 10.1371/journal.pone.0190423

**Published:** 2018-01-03

**Authors:** Nicola Willis, Webster Mavhu, Carol Wogrin, Abigail Mutsinze, Ashraf Kagee

**Affiliations:** 1 Africaid, Harare, Zimbabwe; 2 Centre for Sexual Health and HIV/AIDS Research (CeSHHAR), Harare, Zimbabwe; 3 Department of Psychology, Stellenbosch University, Stellenbosch, South Africa; TNO, NETHERLANDS

## Abstract

**Background:**

Studies have found that adolescents living with HIV are at risk of depression, which in turn affects adherence to medication. This study explored the experience and manifestation of depression in adolescents living with HIV in Zimbabwe in order to inform intervention development.

**Methods:**

We conducted a body mapping exercise with 21 HIV positive 15–19 years olds who had been diagnosed with major depressive disorder. Participants created a painted map of their body to assist them in expressing their somatic and emotional experiences in qualitative interviews. The interviews were transcribed and thematically coded using NVivo 10.

**Results:**

Participants attributed their experiences of depression to their relationships and interactions with significant people in their lives, primarily family members and peers. A sense of being different from others was common among participants, both due to their HIV status and the impact HIV has had on their life circumstances. Participants described a longing to be important or to matter to the people in their lives. A sense of isolation and rejection was common, as well as grief and loss, including ambiguous and anticipated loss. Participants’ idioms of distress included ‘thinking deeply’ (‘*kufungisisa*’), ‘pain’, darkness, ‘stress’ or a lack of hope and ambiguity for the future. Suicidal ideation was described, including slow suicide through poor adherence. Supportive factors were also relational, including the importance of supportive relatives and peers, clinic staff and psychosocial support programmes.

**Conclusions:**

An understanding of HIV positive adolescents’ own narratives around depression can inform the development and integration of appropriate mental health interventions within HIV care and treatment programmes. Study findings suggest that family and peer-led interventions are potentially useful in the prevention and management of depression in adolescents living with HIV.

## Introduction

Depression is the third leading cause of illness and disability among adolescents and suicide is the third leading cause of death in adolescents aged 15–19 years [1]. Although adolescents living with HIV are commonly exposed to multiple risk factors associated with depression in adolescents [2–11], there has been a significant lack of attention to the prevalence, manifestation, impact and management of depression in adolescents living with HIV. Several studies in Zimbabwe and elsewhere have found that adolescents living with HIV are at risk of depression [9, 12–13], which in turn correlates with poor adherence to antiretroviral therapy (ART) [7]. Recent research in Zimbabwe has found high rates of virological failure (viral load ≥1000 copies/ml) among adolescents living with HIV [14]. Despite the global success of antiretroviral treatment programmes and an overall decrease in AIDS-related deaths, mortality rates continue to increase in the adolescent age group [15–16].

International guidelines now call for the integration of mental health within HIV service delivery [17]. Evidence-based, adolescent-focused interventions which prevent and manage depression are urgently needed. If HIV services are to effectively meet adolescents’ needs, it is necessary to understand the experience and manifestation of depression in adolescents. Here we report on a study that explored the experience and manifestation of depression in adolescents living with HIV in Zimbabwe in order to inform intervention development.

## Methods

### Participants

Between January and June 2015, in-depth interviews were conducted with 21 HIV positive adolescents aged 15–19 years and diagnosed with major depressive disorder (MDD). Participants were recruited through purposive sampling from the Zvandiri ('As I am') programme, a model of differentiated clinical service delivery for HIV positive children and adolescents in Zimbabwe (www.africaid-zvandiri.org). In the Zvandiri programme, adolescents are routinely screened for common mental disorders and those at risk are referred to a registered clinical psychologist or psychiatrist for further assessment and management. Adolescents who received the diagnosis of major depressive disorder by the psychologist or psychiatrist using Diagnostic Statistical Manual of Mental Disorders, 4^th^ edition (DSM-IV) [18] criteria were provided with information about the study and invited to participate. All participants were seen within two weeks of their diagnosis of depression. None had been initiated on anti-depressant medication at the time of the interview. Participants were recruited until thematic saturation.

### Procedure

The interviews took place at an adolescent treatment centre in Harare and were structured around a body mapping process, a creative arts technique [19–21]. Participants were asked to create a painted map of their body to assist them in externalizing their somatic and emotional experiences. An interview and body mapping guide was used to facilitate the interview (NW, AM and a research assistant), which aimed to engage the participant in a creative dialogue around the research questions. It contained open-ended questions to explore their subjective experiences of depression and perceptions of care received.

**Fig 1:**
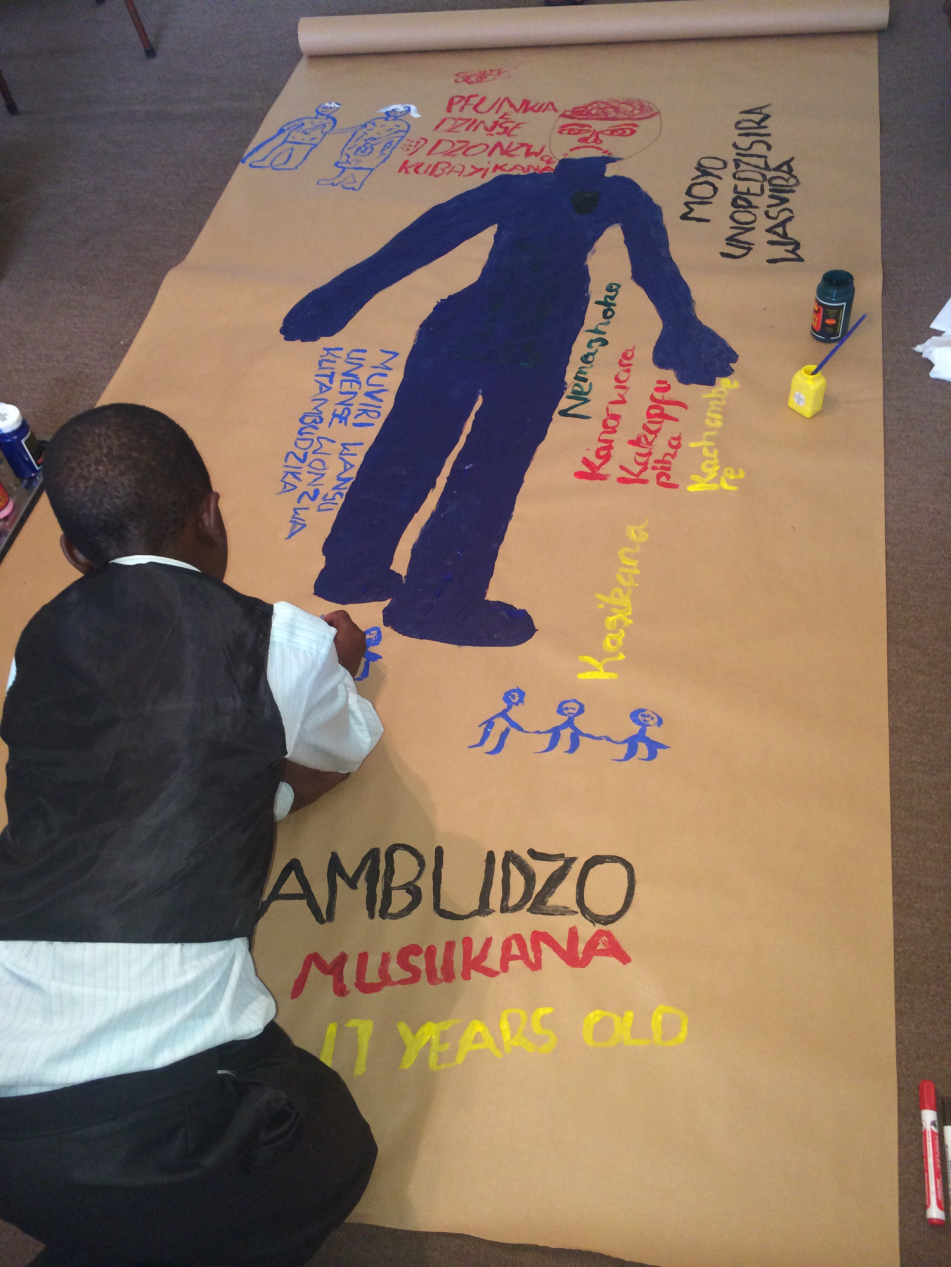
Body mapping session.

At the beginning of the interview, an outline of the participant was drawn on a large sheet of paper. The interview was then conducted and with each question, the participant was invited to add words, colours or pictures to their body map, to assist them in responding to the questions (Fig 1). The questions focused first on socio-demographics (name, age, gender, HIV and ART history), then asked the participant to think about the word "depression", its meaning and how depression has affected them. Depression was not a new word to them as they had all been informed of their diagnosis of depression when assessed by the psychologist or psychiatrist prior to enrolment in the study.

Participants were then asked to focus on what may have contributed to the depression, their thoughts about the future and the type of support they desired and needed. Probes were used to engage the participant in a deeper exploration and to elicit descriptive statements and expressions of their experience and perceptions. Interviews were conducted by professional counsellors who had extensive experience in body mapping as a therapeutic process with HIV positive adolescents. Each interview lasted between 1 and 1.5 hours and was conducted in English or Shona, according to the preferences of each participant. All interviews were audio-recorded.

### Data processing and analysis

Each in-depth interview was transcribed and translated into English, if in Shona, and the body maps were photographed. Pseudonyms were used and any personal identifiers were removed from the transcripts before being entered into NVivo 10 (QSR International, Melbourne, Australia), a qualitative data storage and retrieval program. Verbal and visual data were then separately coded by two individuals (NW and CW) and then compared. Discrepancies were resolved by discussion. Once the coding was consistent for both the transcripts, they were single-coded. Codes were then grouped into categories and emerging themes were identified. Common themes from the different interviews and body maps were then identified and illustrated with quotes and images. The body maps were stored in a locked cupboard which was only accessible by the lead researcher. Once coded, participants were offered their body map but all preferred a photograph rather than a life size original. The original body maps were then destroyed.

### Ethical considerations

Ethics approval was given by the Medical Research Council of Zimbabwe (MRCZ/B/665) and the Human Research Ethics Committee at Stellenbosch University. Prior to participation in the study, written informed consent was obtained from all participants 18 years and above. For minors, written consent was obtained from guardians and written assent was obtained from the participants themselves. Names used throughout this paper are pseudonyms chosen by the participants. Participants were given a stipend for participating in the study as well as being provided with lunch. No current acute suicidal ideation was expressed. However, participants who suggested on-going passive suicidal ideation were referred to a psychologist / psychiatrist at the treatment centre. All participants were offered follow up counselling and all readily consented.

## Results

### Demographic characteristics of participants

Of the 21 study participants, 11 (52%) were male and 19 (90%) were orphans, with 12/19 (63%) having lost both parents, 3/19 (16%) having lost a mother and 4/19 (21%) having lost a father. Only 3/21 (14%) participants lived with a biological parent. The majority of adolescents (18/21, 86%) stated during the body mapping process that they had acquired HIV from their mother whilst the remaining 3 (14%) were horizontally infected with HIV following sexual abuse as children. About three quarters (16/21, 76%) were on first line antiretroviral therapy whilst the remainder (5/21, 24%) were on a second line therapy regimen. Below, we describe the themes that emerged from qualitative data. One adolescent declined to participate in the study.

### Idioms of distress

Participants’ idioms of distress were conveyed through both their verbal narratives and the words and images which they painted on their body maps. The most commonly used terms to describe depression were *‘thinking deeply’ (kufungisisa)* or being ‘*lost in thought’* as result of the events in their lives. ‘*Darkness*’, ‘*pain*’, ‘*stress*’ and *‘hopelessness’* were also common.

Suicidal ideation was commonly referred to, including ‘slow suicide’ by five participants who described a desire to intentionally default on their antiretroviral treatment as a means of ending their lives. One female participant stated *“Sometimes I feel like giving up on life by not taking my medication [antiretroviral] so that people stop talking about my status [HIV]” (Melissa*, *18 years)*.

Some participants also referred to somatic symptoms of stomach ache and headaches but this information only emerged after probing.

### Contributing factors

Participants described a range of negative and traumatic experiences contributing to their depression. They generally attributed their experiences of depression to the expressed thoughts, behaviours and attitudes of the most significant people in their lives, specifically their relatives and peers. This was conveyed through both their verbal narratives and the evocative imagery which they chose to paint on their body maps to illustrate their experiences and emotions.

Seven main themes emerged from the data which all related to their emotional experiences of depression:

#### 1. Being different from others

Participants commonly described feeling different from others due to their HIV status, particularly in relation to their parents, guardians or siblings they lived with and were HIV negative. They narrated their distress at being physically different (due to stunting and delayed puberty), orphaned and failing at school. They referred to the confusion and pain they felt as a result of being the only one to be HIV infected, asking “*How did I get it*?*”* and “*Why me when others do not have it*?” One boy stated his use of the colour red *“represents the pain that I go through each day I go to school because of my body…It’s small so people always tease me” (Persevere*, *18 years)*.

#### 2. Learning of their HIV status

Participants described feelings of ‘*sadness’*, *‘stress’ and ‘great pain’* in their lives at the time of learning their HIV status, as articulated by one female participant. *“After hearing* (the) *results … when you are told that you have it (HIV)*. *That is when you find all the stresses*. *That is when you start to think of bad ideas… that’s when depression comes” (Tarisai*, *18 years)*.

#### 3. Isolation and rejection

A sense of isolation and rejection was common, whether by others, self-imposed or anticipated. Participants commonly described being ridiculed, laughed at, talked about and excluded by peers as a result of their HIV status or the fact that they took medicines. A female participant also referred to the concerns of her peers around HIV transmission when she explained “*They will not play with me because if they touch me*, *they will be "infected"*. *(Paradzai*, *15 years)*. Participants commonly drew themselves playing alone.

They described their ‘pain’ and ‘hurt’ as being worse when the isolation or rejection in their lives was imposed by the people they expected should support them, and commonly depicted this with paintings of relatives and peers. A male participant stated *“They hurt me when they say*, *'This one drinks pills (for HIV)*, *so there is no need to bother with him because he is not my child'*. *(Wangaa*, *17 years)*.

Some participants referred to the way in which they isolated themselves from others in order to conceal their HIV status and their fears of what would happen if they were to be rejected. One male participant stated “*You will not be drinking the tablets on time*, *because you will be saying*, *'If l drink the tablets and people see me*, *they will laugh at me' (Tambudzai*, *18 years)*.

#### 4. Loss and grief

As stated earlier, the majority of respondents had been orphaned and demonstrated signs of profound, unresolved grief. Participants commonly described the death of a parent/parents as contributing to their depression and expressed a yearning to have known their parents through their words, paintings, body language and emotions. *“You need that love*, *but you won’t get it*. *I cry myself to sleep every day*. *My mother died when l was three*. *I never knew her*, *l don’t have a picture of her” (Janet*, *19 years)*.

Grief was often exacerbated where participants were living in unsupportive households or where a loving, caregiver relationship was lacking. A female participant symbolised her stepmother by drawing a snake and going on to explain, “*I stay with my stepmother who is very cruel to me*. *She ill-treats me because she has her own child and so it is done on the basis that I am not her child*. *My mother passed away a long time ago…I wish she was around” (Paradzai*, *15 years)*.

#### 5. Low self-worth

Participants described a longing to be important or to matter to the people in their lives, specifically family members and peers. Whilst a few described elements of support from caregivers, the majority narrated traumatic relationships with their primary caregiver who lacked care or concern for them compared with their HIV negative siblings or other children in the household. Others stated that they were moved from household to household, without being cared for or loved. One male participant explained *“When I was sick*, *some relatives would say I was to be left to die just the same way my mother died*. *I was moved from relative to relative" (Kudzanai*, *17 years)*.

#### 6. Lack of protection

Participants reported that they had not been protected by the people closest to them, who not only failed to protect them, but also inflicted abuse. A sense of betrayal and disillusionment was evident in their narratives, particularly by those who had been sexually abused. They castigated both the perpetrator and family members who did not seek justice against those who had abused them. One female participant painted her entire body black and explained, “M*y mother died…My father l have but he doesn’t care about me*… *He was sexually abusing me (Janet*, *19 years)*.

#### 7. The future

Most participants expressed high expectations for a successful, brighter future, illustrated by drawings of getting married, having children, achieving academically and gaining employment. The need for independence from unsupportive caregivers was significant for some participants and was linked to the desire to be employed and economically stable. They also stated that marital status and having children of their own, along with completing their education and becoming employed, would improve their sense of self-worth. However, these expectations were often accompanied by an uncertainty regarding whether they could actually achieve these, given their poor health and lack of finances, academic qualifications and support. One male participant stated, “F*or now there is nothing [in his future]*. *It’s just hazy and a bit complicated to understand where it’s headed to*. *At times it’s just sorrowful and just so sad"* (*John*, *18 years*).

Another participant referred to his fear of the future when he painted a hen as a symbol for ‘depression’. He described himself as a ‘hen’ *“because anytime it can be killed*. *So just like me*, *l am like a hen*. *l don’t know when l will die but I just know that l will die because of the situation that l am in” (Prince*, *16 years)*.

#### Supportive factors

A range of factors were identified by participants as supporting them with depression. Participants who had accessed peer-led and psychosocial support services through the Zvandiri programme stated that this support had played a central role in improving their sense of self-worth and confidence, and reducing their sense of isolation and rejection. This was particularly noted among those who lacked support from caregivers or peers in their daily lives. Supportive counsellors were also highlighted by some participants as being important in their care. Educational assistance and skills training for future employment were identified as being critical interventions for their well-being. Participants described feeling relieved as a result of being able to share experiences they had not shared before.

## Discussion

The results from this study confirm the profound significance of family and peer relationships in the lives of HIV positive adolescents with depression. Although learning their HIV status was significant, participants commonly attributed their negative experiences and subsequent depression to the expressed thoughts, behaviours and attitudes of the most significant people in their lives.

Stable, supportive families are critical in promoting normal childhood development [22–23] and there is a strong causal relationship between parental relationships and adolescent depression [13]. Yet the results of this study suggest that stability and support from parents were critically lacking in participants’ lives, with 63% being double orphans and only 14% living with a biological parent. Participants were coping with complicated grief [24], from losses that included not only the death of one or both parents, but siblings and grandparents, as well as the consequences of those losses. Participants reported that they yearned to be loved, accepted, valued and supported by their immediate relatives and the absence of this further compounded their sense of grief and loss for their biological parents. Interventions to support caregivers have been found to improve the mental health of children living with HIV [25, 26] and could be adapted and scaled up for adolescents.

The role of grief and loss in their narratives of depression concurs with the literature indicating that children who experience the death of a parent demonstrate lower self-esteem and higher rates of psychological problems than non-bereaved children [27,28]. Multiple losses can negatively affect the development of a sense of self-worth, interfere with a person’s ability to trust or depend on others, and can lead to an avoidance of close interpersonal relationships [29]. The results from this study and the literature suggest that there is a critical need for therapeutic grief interventions for children and adolescents living with HIV. Yet grief and loss have been largely neglected in this group of young people in Sub-Saharan Africa, despite the magnitude of losses in the lives of children and adolescents living with HIV in the region. A grief intervention for adolescent girls in South Africa was found to significantly reduce the incidence of complicated grief and depression [30]. This intervention model could be replicated for adolescents living with HIV.

The study results also demonstrate the critical role of peer relationships on the confidence, self-esteem and identity of HIV positive adolescents with depression. Participants demonstrated that in their own constructed realities, they felt worthless, of no value and thought they had no future when compared with their peers. Participants expressed a yearning to be accepted and valued by their peers and to have opportunities to socialise with them, and to also identify with them physically, socially and developmentally. They also commonly referred to the way in which their peers do not need to take medication. In the same way that peers were identified as contributing to their negative experiences, participants clearly described them as being central to the support that they required.

Adolescence is a unique stage of life characterised by rapid growth and development and characterised by increasing autonomy, independence and an intense desire to associate and identify with peers [11]. Yet the participants described multiple challenges which they perceived as making them different from their peers. Skin disfiguration, stunted growth and pubertal delay were clearly identified as contributing to stigmatising behaviour by peers, setting them aside from their peers and affecting their confidence and self-esteem. A fear of peers or partners finding out their HIV status was common in all narratives. Participants also described being different due to poor academic achievement in school and narrated a deep yearning to succeed, like their peers. Although skin disfiguration, growth and pubertal delay, cognitive impairments and fear of disclosure to others are described in the literature [6, 31–32], there has been a lack of attention to the impact on adolescents’ mental health.

The World Health Organisation now recommends peer-led interventions for adolescents living with HIV [17]. Several models of group-based [33–35] and peer-led interventions [14] exist for adolescents living with HIV and there is some evidence that these have contributed to improved retention, psychosocial well-being and virological suppression. However, studies that specifically investigate the role of HIV positive young people in supporting their peers diagnosed with depression are lacking. Lay counsellors have been found to be effective in addressing the mental health of adolescents living with HIV [26] and there is now need to investigate the effectiveness of adolescents and young people in this role [36]. Similarly, despite the growing number of studies that show HIV-infected adolescents are at increased risk of mental health problems including depression, current models of adolescent HIV service delivery worldwide do not integrate mental health services. This lack of attention to the importance of mental health services for young people with HIV leads to challenges in ensuring early, accurate diagnosis and treatment, resulting in the mental health needs and HIV treatment and care being unmet. This is not only essential for their own HIV outcomes but also for their development, and survival in to adulthood.

There is need to ensure that national HIV policies and guidelines are inclusive of mental health and that health care workers are trained to respond to the multi-faceted mental health needs of adolescents living with HIV. Since most sub-Saharan African countries (Zimbabwe included) have a critical shortage of psychiatrists [37], it is important that mental health interventions be adapted for health workers and lay workers so that mental health services can be effectively rolled out and integrated within the national HIV programme. This approach has been effectively implemented in Zimbabwe by the Friendship Bench which engages lay counsellors as therapeutic counsellors for adults with depression [38]. Also in Zimbabwe, HIV positive adolescents and young people trained as Community Adolescent Treatment Supporters (CATS) have been found to be effective in improving adherence and psychosocial well-being among their HIV positive peers [39, 40] and current studies are investigating their effectiveness in improving common mental disorder in adolescents living with HIV [14].

A potential limitation of this study is that the sample size was small and it may therefore not be possible to make generalizations about the larger population of adolescents living with HIV and depression. However, these in-depth data from young people in urban Zimbabwe provide important evidence towards an improved understanding of the needs and experiences of HIV positive adolescents with depression and their perceptions of the care they have received.

## Conclusions and recommendations

An understanding of the narratives of depression among adolescents living with HIV is necessary to inform the development of services which are responsive to their mental health needs, and can have a positive impact on the long-term HIV treatment and care outcomes, and transmission risk to partners and children. The findings from this research suggest that family and peer-led interventions may be effective in preventing and responding to depression among adolescents living with HIV and should be a key component of differentiated service delivery models for this age group, in order to improve their mental health as well as adherence to ART. Efforts should focus on the development and scale up of family interventions and equipping peer counsellors with skills to integrate mental health interventions within their work with adolescents living with HIV. However, studies are needed to evaluate the effectiveness, acceptability and feasibility of such family and peer-led mental health interventions in preventing and managing depression and improving adherence to ART.

## Supporting information

S1 File Minimal data set. (DOCX)

S2 File COREQ checklist. (PDF)

S3 File In depth interview and body mapping guide. (DOCX)
