# Supplementary material for: Understanding the experience and manifestation of depression in adolescents living with HIV in Harare, Zimbabwe
Source: PLoS One. 2018 Jan 3;13(1):e0190423. doi: 10.1371/journal.pone.0190423 (PMC5752002; doi:10.1371/journal.pone.0190423)
Supplement: S1 File — (DOCX) [file pone.0190423.s001.docx]

| **Understanding the experience and manifestation of depression in adolescents living with HIV in Harare, Zimbabwe**  **Willis, N., Mavhu, W., Wogrin, C., Mutsinze, A., Kagee, A** | | |
| --- | --- | --- |
| **Theme** | **Sub-theme** | **Quotes** |
| **SUBJECTIVE EXPERIENCES OF DEPRESSION** | | |
| Being different from others | HIV Status | “*Why me when others do not have it*?”  “*HIV…how did l get it? My father does not have it and it is said my mother did not have it. ….Up to now it still depresses me. My brother and my dad don’t have it”*  “*Why did you choose me, meaning l am no longer a human being? I am just someone that is not the same as others”*  *“My friends at all times are the first cause of my depression. Why? Because what they are and what l am are totally different”*  “*I don’t want anyone to see me drinking tablets, because if l was seen drinking they would laugh at me or they would talk about me or the person would stop playing with me because l drink tablets”* |
|  | Physical differences | “*My* *body is hurt by the words said by people that I am sick, I am short”*  *“Red represents the pain that I go through each day I go to school because of my body…it’s small so people always tease”* |
|  | Orphanhood | *“My body will be depressed when people pass comments. Am I not a person if I’m being looked after by my grandparents?”*  *“Judged*’ or “*looked down upon”*  *“The reddish at the bottom is how I feel sometimes when I am lonely. When I think sometimes of my late father. Because there is my uncle….he has a son. Sometimes when I look at them, they talk, they understand each other. I always wish I had a father.”* |
|  | Educational Failures | *“The inability to read and write. It is what affects me, because you can see a grade two child taking a book and being able to read but l am not able to such that it is something that pains me”* |
|  | Poverty | *“If* *l cannot find just a little amount to go to school then l am not anything in this world so l was depressed”* |
| Learning of their HIV status |  | *“After hearing the (HIV) results … when you are told that you have it (HIV). That is when you find all the stresses. That is when you start to think of bad ideas…. that’s when depression comes”*  *“I was greatly pained….. I knew then that the old me I knew was dead and replaced by a new person residing inside of me”* |
| Isolation and rejection | Isolation and rejection by others | *“They will not play with me because if they touch me they will be infected”*    *“When I was sick some relatives would say that I was to be left to die just the same way my mother died. I was moved from relative to relative ….. It hurt, but then I had no one to share with”*  *“It is worse when it comes from someone whom you think is there to support you but rather he is the first one to bring you down”*  *“They hurt me when they say this one drinks pills, so there is no need to bother with him because she is not my child”* |
|  | Self-imposed isolation | *“I am so scared to keep the tablets in my bag (*when I go on camp*). I am hiding, I am protecting myself*”  *“I find it difficult having friends because they will disclose my status and talk about me behind my back. They will start looking down upon me, and so that is why I prefer doing things alone”* |
|  | Anticipated isolation and rejection | *“Being told…”this is not your home, you will leave anytime”*  *“I wish I could just tell her (my girlfriend) [my status] and she would understand but the way if I tell her the other thought is like if I tell her what will she think about me?”*  *“You will not be drinking the tablets on time, because you will be saying if l drink the tablets and people see me they will laugh at me”* |
| Loss and grief | Actual loss and grief | *“You need that love, but you won’t get it. I cry myself to sleep every day. My mother died when l was three. I never knew her, l don’t have a picture of her”*  *“I think if my mother was around she would be beside me, talking and just doing things that mothers and children do. I still want to think of my mom, I just don’t want the thoughts of her going…. (but) when I think of my mum, I don’t do good, I don’t feel well mostly”*  *“I stay with my stepmother who is very cruel to me. She ill-treats me because she has her own child and so it is done on the basis that I am not her child. My mother passed away a long time ago….I wish she was around”*  *“All I could do was cry and blame my mother because she had just disappeared on me…. how could my mother do this to me?”* |
|  | Ambiguous loss and grief | “*I lived a life where l did not know where my father was, where my mother had gone to. I grew up with my maternal grandmother. That’s why l am saying it was all darkness”*  *“If l know my father, and if l know if he is alive or not, l think that is what would make me happy for now”* |
|  | Anticipated loss and grief | “W*hat if she dies? What will happen? Yet she is the one with information about who my mother dated and stuff like that”* |
| Low self-worth |  | *“When I was sick some relatives would say that I was to be left to die just the same way my mother died. I was moved from relative to relative”*  “…*(my brother) is HIV negative so when l compare him with me l just realise that l may not be that loveable…because he is learned, he has a good job and all that”. But l don’t have anything”*  *“I am like a pawn in a chess set… a pawn is worthless among other pieces”*  *“I regret being born. I blame myself for my mother falling pregnant and having to conceive me”* |
| Lack of protection |  | “*the way the people in my life have ill-treated me*”  “m*y mother died…..my father l have but he doesn’t care about me. ….. He was sexually abusing me”*  “*I will be wishing* (they) *would come off me, but you find them all there. ...Like the things of mischief and naughtiness (*the rape*) l was talking about, you will be wishing if they would leave me. The black l have drawn there reflects the people who want to hurt me. Those who want to rape and sleep with me”* |
| The future |  | “*l should be a graduate and l should be a worthy person among other people*”  “*I wish that I could be able to be someone in life and I wish to be able to there for my son, my daughter in the future”*  *“This makes me feel depressed cause it’s what I want to be in life.….so sometimes the disease that I have makes me feel sad”*  “F*or now there is nothing (*in my future*). It’s just hazy and a bit complicated to understand where it’s headed to. At times it’s just sorrowful and just so sad*”  *“It doesn’t bring happy thoughts to me so l don’t think about my future”*  “I drew myself as a hen *because anytime it can be killed. So just like me l am like a hen. l don’t know when l will die but I just know that l will die because of the situation that l am in”* |
| **IDIOMS OF DISTRESS** |  |  |
| Thinking too much |  | “kufungisisa” |
| Stress |  | *“I think depression is when a person is under stress and it’s this stress which in turn leads them to doing the unexpected such as committing suicide because they would have given deeper thought of what is affecting them in their life and this troubles them”* |
| Challenges with daily functioning |  | *“It’s more like everything I do I have to work hard. There is no like easy time. Even when I am sleeping it’s not easy”*  “D*epression, I think is something that doesn’t want you to achieve something that you want as it keeps on keeping you down”* |
| Pain |  | *“Pain is when sometimes I feel sick cause sometimes like each year there is not a year I was normal. I go through every day with the suffering of knowing who I am and knowing that I cannot change anything”*  *“My left hand, I also painted black because I felt a lot of pain (there). I have held bad things and what I have felt with my hands that are bad that deeply depresses me”*  *“l am just a person who by when people see me smiling…it’s a fake smile. l don’t smile but l hide all the pain that l feel inside and smile all the time”* |
| Darkness |  | *“My heart is dark*”  *“The whole of my head is full of darkness. Let us say my thoughts and things I have seen or things I have said they are all bad. What I have faced is full of darkness that is why I painted my head black”*  *“Black shows that there is no light that l am seeing or brightness ahead of me”* |
| Hopelessness |  | *“l don’t see a future for myself at times. l feel it’s better to die, there is no reason to live”*  *“It’s like l don’t have life, it’s just me in my own world. I have no hope for the future that l will be someone. Right now l have dropped out of school ........ Sometimes l feel l should just die”*  *“It has made me feel like not possible in life, it has made me feel like giving up and do drugs, alcohol and those kinds of things”* |
| Suicidal ideation |  | *“I was disappointed to know I was HIV positive and I contemplated being dead was better”*  *“On my neck I painted a rope because there are times when I feel like killing myself using a rope, just hanging myself. Then on my head I drew small circles that are representing tablets. Sometimes I feel like committing suicide so that’s why I used them”*  *“Sometimes I feel like giving up on life by not taking my (*antiretroviral*) medication so that people stop talking about my (*HIV*) status”* |
| **PERCEPTIONS OF CARE** |  |  |
| Families |  | “*Yhe fact that she works hard to feed us, and (provide) clothes … gives me a reason to move on, and sometimes it depresses me that she is no longer able to do what she used to do when she was still very active to take care of us”*  *“He* “*constantly tells me that I am a sick person”*  *“That is what l wish for as a child. l wish if my mother was there. So l wish if my sister would take that place to help…* i*t is good if she would just visit and l will see that she cares* |
| Peers |  | *“I feel like I have a lot of people around me but to understand me? They do not understand me. That is how I feel”*  *I have drawn myself in the past when l was alone but as time progressed l got support from my peers then l saw that I have a bright future”*  “(In the support group) *I received love that I was not getting from my relatives. I now have a friend from my support group which I am glad because at my school there was stigmatization”* |
| Service Providers |  | *“What helped me not to be depressed was the fact that l went to the doctors to ask them what had happened, that’s when l stared accepting it”*  *“That gave me hope to living…they introduced me to support groups, where I would mingle with other HIV positive children like me”*  *“They love me at Africaid*”.  *“Zvandiri House has always been there for us. Even the counsellors telling us to accept who we are and helping us to talk about the things that we feel, and how we can find solutions to help us get better and to help us to the level we want to get. I will draw the aunts and uncles and the House as well. I feel that if they had not been there in my life things could have been very complicated than they are. They were difficult for me, I would still be that unhappy child..* *they are those people in my life to guide me and support me and to encourage me in my life, showing that I am needed, that I am great, important to the society”*  “*Because l know l am going to get support, l know l am not carrying this alone, so my road is easier to walk on”* |
