# Supplementary material for: Understanding the experience and manifestation of depression in adolescents living with HIV in Harare, Zimbabwe
Source: PLoS One. 2018 Jan 3;13(1):e0190423. doi: 10.1371/journal.pone.0190423 (PMC5752002; doi:10.1371/journal.pone.0190423)
Supplement: S3 File — (DOCX) [file pone.0190423.s003.docx]

## **In-depth interview and body mapping guide**

**Research Study: Subjective experiences and perceptions of care among depressed adolescents living with HIV attending a community adolescent HIV programme.**

**IN-DEPTH INTERVIEW AND BODY MAPPING GUIDE**

**Step 1: Getting Started**

Welcome the participant to the interview and body mapping session. Focus on ensuring the participant feels safe and comfortable.

Review the participant’s consent and assent forms, asking the participant to confirm the signatures on the forms.

Remind the participant that this process is completely voluntary and if he/she wishes to stop at any time, then he/she may do so without any implications on his/her future care.

Remind the participant that you are going to have an interview which will focus mainly around the development of a body map. Ask the participant if he/she has any questions before you get started.

**Step 2: Body Mapping Guide**

1. Start by giving the participant a large piece of paper and laying it down on the floor. Explain to the participant that you will now trace the outline of his/her body. Ask the participant to lie down on the paper and with a black marker pen, trace around the participant’s body. When the participant stands up, explain that this represents his/her body and that he/she will now be exploring his experiences living with depression by adding colour, pictures, words and symbols to the body map. Emphasize that this is not a test of his/her artistic skills and ability.
2. Start by asking the participant to choose a colour which he/she feels represents him and paint his body using that colour. Ask the participant to explain why he/she chose that colour.
3. Now ask the participant to write the following on the edge of his/her map: age, gender, who he/she lives with, how long he/she has known his/her HIV status and ART history. Explain that he/she can use colours, drawings, words and symbols.
4. Remind the participant that the purpose of this study is to explore the participant’s experiences of depression and perceptions of care. Ask the participant to think about the word ‘depression’. Ask him/her to think of a symbol which represents ‘depression’ for him/her and to draw this on the body map. Explain that he/she can choose where he/she thinks is the most appropriate place to put this. Ask the participant to then explain why he/she chose the symbol, its meaning and positioning on the body.
5. Now ask the participant to reflect on how depression has affected him/her? If necessary, use the probes “how have you been feeling over the past two weeks?” what have you been thinking about over the past two weeks?” Additional probes may be asked about appetite, sleep, energy, interest and pleasure, concentration and optimism. However these probes should be limited where possible to minimize demand characteristics. Ask the participant to explain what he has drawn or written.
6. Now ask the participant to reflect on what he/she thinks may have contributed to this depression? What may have caused it? Explain to the participant that he/she can choose where to place these drawings or words. It may be on the inside of the body or on the outside. Ask the participant to explain the words and drawings and their positioning on the body map.
7. Now ask him/her to describe how he/she thinks and feels about his/her future? Ask him/her to use words, symbols and colours on the map to symbolise this and to explain what he/she has written or drawn and the positioning on the map.
8. Now ask the participant to think about whether he/she has been getting any support? Has this helped with the thoughts, feelings and behaviour he/she has been describing? Ask him/her to use words, symbols and colours to show what support he/her has been getting and how it has helped? Ask him/her to explain this and the type of support, who was providing it, where?
9. Now ask him/her to use words, pictures and colours to show what else would make him/her feel better? Ask him/her to explain what he/she has written or drawn and the positioning on the body map.
10. Ask the participant whether there is anything he/she would like to add to his/her body map that he may have forgotten or that he/she feels has been missed.
11. Ask the participant to look at his completed body map and ask how he/she feels when he/she looks at his/her body map. Then ask how he/she felt during the process of making the body map.
12. Ask the participant if he/she would like a photograph of their body map to keep. If the participant would like a photograph, photograph the body map and inform that he/she will be given the photograph after two days when it has been printed. Remind the participant that the body map will be kept confidentially in a locked cupboard and that only the researcher (NW), research assistant and supervisor (AK) will have access to the body maps. However, as explained in the consent and assent form, words and pictures from the body map may be used in the research report but these will be anonymous with no identifying markers.

Thank the participant for his/her time and participation in the research.
